# Synbiotic effects of 2’-fucosyllactose and *Bifidobacterium longum* subsp. *infantis* M-63 in fermented human fecal communities

**DOI:** 10.3389/fnut.2026.1744839

**Published:** 2026-05-14

**Authors:** Shijir Xijier Mingat, Tatsuya Ehara, Hirohisa Izumi, Ryuta Ejima, Eri Mitsuyama, Hirohiko Nakamura, Kazuhiro Miyaji, Jin-zhong Xiao

**Affiliations:** 1 Health Care & Nutritional Science Institute, R&D Division, Morinaga Milk Industry Co., Ltd., Zama, Kanagawa, Japan; 2 Biotics Research Institute, R&D Division, Morinaga Milk Industry Co. Ltd., Zama, Kanagawa, Japan; 3 Morinaga Milk Industry Co. Ltd., Tokyo, Japan; 4 Department of Microbiota Research, Juntendo University, Graduate School of Medicine, Tokyo, Japan

**Keywords:** 2’-fucosyllactose, *Bifidobacterium longum* subsp. *infantis* M-63, gut microbiome, human milk oligosaccharides, human-residential bifidobacteria, infant formula and young child formula, synbiotics

## Abstract

**Background/objectives:**

Human milk oligosaccharides (HMOs) are the third most abundant solid component of human milk. HMOs are selectively utilized by infant-type human-residential bifidobacteria (HRB), resulting in the formation of a gut microbiota dominated by bifidobacteria and the production of health-beneficial metabolites, such as acetate and aromatic lactic acids (ALAs), in breastfed infants. HMOs play key roles in infant health by acting as prebiotics, preventing infections, and regulating the immune system. However, the prevalence of HMO-utilizing bifidobacteria in the gut microbiota of infants and young children varies greatly between countries and regions, with some infants and children containing none.

**Methods:**

We used a pH-controlled single-batch fermenter to model the human gut microbiota and evaluated whether HMOs provide infants or young children having or lacking bifidobacteria with HMO-utilizing ability with any physiological benefits. We conducted fecal fermentation with 2’-fucosyllactose (2’-FL), with or without supplementation with a probiotic HRB strain (*Bifidobacterium longum* subsp. *infantis* M-63).

**Results:**

2’-FL alone did not significantly increase the relative abundance of bifidobacteria or the production of acetate and ALAs during fecal fermentation of infants and young children. Conversely, 2’-FL + M-63 significantly increased bifidobacteria and promoted acetate and ALA production in the fecal fermentation of both infants and young children.

**Conclusion:**

Health benefits from 2’-FL may be restricted by inter-individual and age-dependent differences in gut microbiota response. Supplementation with a probiotic HRB with high HMO-utilizing ability could overcome this restriction. Our findings provide insights into the development of formulas for infants and young children.

## 1 Introduction

Human milk oligosaccharides (HMOs) are the third largest (10–20 g/L, after lactose and lipids) solid component of human milk and highly diverse, with over 200 types identified (1, 2). HMOs reach the colon without being digested in the human digestive system and have been reported to exhibit beneficial physiological effects, such as modulation of the gut microbiota in both infants (3–5) and healthy adults (6), prevention of gastrointestinal infections (7), suppression of intestinal inflammation (8), and development of brain functions via the potent gut–brain axis (9). Therefore, differences exist between breastfed and formula-fed infants, and HMOs, which are not included in traditional infant formulas, may serve as candidates to bridge this gap. Although it is challenging to manufacture all HMOs due to their complex structures, recent technological advancements have enabled the production of several types (10, 11). Currently, the addition of HMOs—especially 2’-fucosyllactose (2’-FL), one of the most abundant representative HMOs in human milk—to infant formula is becoming a global trend (10–12). Furthermore, recently, the addition of HMOs to formula for children aged 3 year and older has also begun.

HMOs are utilized by infant-type human-residential bifidobacteria (HRB), such as *Bifidobacterium bifidum, B. breve, B. longum* subsp*. longum (B. longum)* and *B. longum* subsp. *infantis (B. infantis)* (13, 14). During this process, HRB produce metabolites, such as acetic acid and aromatic lactic acid (ALA) (15, 16). These metabolites have been reported to benefit the host by preventing infections, metabolic and immune regulation, and anti-inflammatory effects (15, 16). At least some of the effects of HMOs are supposedly mediated through the production of these metabolites by HRB (15, 16).

Among infant-type HRB, *B. infantis* has a particularly high utilization ability of HMOs. *B. infantis* promotes vaccine reactivity (17), improves malnutrition (18), and prevents necrotizing enterocolitis (19), suggesting its potential beneficial effects on infant health. Conversely, the prevalence of *B. infantis* in the gut microbiota of infants varies greatly by country and region, and it is often infrequently or hardly detected in industrialized countries (20). Furthermore, infant-type HRB, including *B. infantis*, decrease around the time of weaning, transitioning to an adult-type gut microbiota by about 3 years of age (21, 22). However, sufficient verification has not been conducted on whether HMOs can exert their expected effects in infants who do not harbor *B. infantis* in their intestines, or in children aged 3 years and older after *B. infantis* has declined. It has also been suggested that for such populations, supplementation with bifidobacteria, especially *B. infantis,* as probiotics in combination with HMOs may be necessary (23).

*B. infantis* M-63 is a probiotic strain with high HMO utilization ability that exhibits potential to improve gastrointestinal conditions via acetate and ALA production (23). In infants administered M-63, the number of bifidobacteria in the intestine correlates with the frequency of breast milk intake, suggesting that at least some of the effects of *B. infantis* are related to the utilization of HMOs from human milk (24).

In this study, we investigated the effects of HMO alone or in combination with *B. infantis* M-63 on the gut microbiota of infants within 7 days of age and children aged 3–5 years, specifically focusing on the prebiotic effects using a fecal culture system. We also evaluated changes in the production of HRB-associated metabolites such as short chain fatty acids (SCFAs), lactate, and ALAs. We selected 2’-FL as the HMO as it is one of the most abundant HMOs in human milk and is the most commonly used in infant and young child formulas (1, 2).

## 2 Materials and methods

### 2.1 2’-FL and *B. infantis* M-63

*B. infantis* M-63 was provided by Morinaga Milk Industry Co., Ltd. (Tokyo, Japan). 2’-FL (>91%) was supplied by Kyowa Hakko Bio Co., Ltd. (Tokyo, Japan).

### 2.2 Collection of fecal samples

This study was conducted in accordance with the Declaration of Helsinki and was approved by the Japan Conference of Clinical Research (protocol code 101–034, approval date: August 18, 2023), and written informed consent was obtained from the parents. We recruited four healthy infants aged up to 7 days and four young children aged between 3 and 5 years. Fecal samples were collected from each participant at the time points indicated in Table 1 and stored at −25 °C for a few days (for infants) or <10 °C within 8 h (for young children), under anaerobic conditions using AnaeroPack (Mitsubishi Gas Chemical Co., Ltd., Tokyo, Japan). The fecal samples were then diluted 10 times with saline and stored at −80 °C until the experiment.

**Table 1:** Age and species of infant-type HRB in the gut microbiota of the participants.

Participant ID	1	2	3	4	5	6	7	8
	Infant				Young child			
Age	5 days	7 days	7 days	7 days	3 years 0 month	3 years 5 months	4 years 2 months	4 years 9 months
*B. longum*	+	+	+		+	+	+	+
*B. breve*	+		+	+	+	+	+	+
*B. infantis*								
*B. bifidum*				+	+			
*B. adolescentis* group							+	
*B. catenulatum* group					+	+	+	+

Species detected by quantitative real-time PCR are indicated with the symbol “+”.

### 2.3 *In vitro* fecal fermentation

Fecal fermentation was carried out using a pH-controlled multichannel jar fermenter (Bio Jr. 8; ABLE Co., Ltd., Tokyo, Japan), following the procedure outlined in a previous study (25). Briefly, 2’-FL (1% w/v) and M-63 (10^8^ cells/mL) were added to the yeast extract, casitone, and fatty acids (YCFA) medium, followed by addition of a 100 μL aliquot of a fecal sample (containing 10 mg of feces). After 24 h of cultivation at 37 °C under anaerobic conditions, the culture media were collected and centrifuged. The supernatant was used for metabolite measurements, and the bacterial cell pellets were subjected to DNA extraction.

### 2.4 DNA extraction

DNA was extracted using the bead-beating method as described previously (25). Purification was performed using a GenePrep Star PI-480 automatic DNA extraction machine (Kurabo Industries Co., Ltd., Osaka, Japan). Bacterial DNA was subjected to quantification of bacterial cell numbers by quantitative real-time PCR and analysis of microbiota composition using 16S rDNA gene sequencing.

### 2.5 Quantification of bacterial cell number

*Bifidobacterium* species numbers were determined through real-time PCR as described previously (25). The primers and PCR conditions are detailed in Supplementary Table S1 (26–28).

### 2.6 Analysis of the 16S rRNA gene

DNA was amplified as previously described (29). The V3-V4 region of the bacterial 16S rRNA gene was sequenced using the paired-end method on the Illumina NextSeq 1,000 platform using the NextSeq 1000/2000 P1 reagent kit (600 cycles) (Illumina, San Diego, CA, United States). Sequences were analyzed using QIIME2 (version 2022.8) (30). The demultiplexed reads were processed in several steps: filtering, denoising, merging, chimera removal, and the generation of amplicon sequence variants using DADA2 (31). Amplicon sequence variants were taxonomically assigned based on the Greengenes2 database (version 2022.10).

### 2.7 SCFAs and lactate analyses

The quantities of SCFAs and lactate produced during cultivation were calculated by subtracting the concentration at 0 h from those at 24 h. Each culture was centrifuged at 8,000 × g for 10 min at 4 °C, and the supernatant was filtered through a 0.22 μm membrane filter (TORAST Disc NYLON membrane; Shimadzu Co., Ltd. Kyoto, Japan). After appropriate dilution, the samples were subjected to high-performance liquid chromatography (HPLC; Nexera X2 system, Shimadzu). HPLC analysis was conducted as previously described (29).

### 2.8 Analysis of ALAs

To remove high-molecular-weight substances from the culture supernatant, it was mixed with an equal volume of methanol containing the internal standard 1-Methyl-2-oxindole (CAS 61–70-1). After centrifugation at 8,000 × g, 4 °C for 5 min, the supernatant was filtered through a 0.22 μm syringe filter PVDF membrane (FJ13BSCPV002AL01, GVS S.p.A., Bologna, Italy) to prepare the sample for measurement. Liquid chromatography–tandem mass spectrometry (LC–MS/MS; LC–MS-8045 Shimadzu) analysis was performed as described previously (29).

### 2.9 Analysis of 2’-FL

The concentration of 2’-FL in the culture supernatants at 0 and 24 h was measured following the same procedure used for SCFAs and lactate analysis. The supernatant was appropriately diluted, and subjected to LC–MS/MS analysis as described previously (32). For pretreatment, an equal volume of acetonitrile (KOKUSAN CHEMICAL Co., Ltd., Tokyo, Japan) was added to the sample and vortexed thoroughly. The mixture was then centrifuged at 21,500 × g for 15 min at 4 °C. The supernatant was filtered through a 0.22 μm syringe filter PVDF membrane (FJ13BSCPV002AL01, GVS S.p.A.). The filtrate was diluted with 50% acetonitrile before being subjected to analysis.

Ultra-HPLC separation was achieved using an ACQUITY UPLC Glycoprotein BEH Amide Column (1.7 μm, 2.1 × 150 mm, Waters, Milford, MA, United States) on a Vanquish Flex Ultra-HPLC system (Thermo Fisher Scientific, Waltham, MA, United States). The gradient elution solvent consisted of buffers A (10 mM ammonium formate with 0.1% formic acid) and B (0.1% formic acid in acetonitrile). The flow rate and column temperature were set at 0.35 mL/min and 35 °C, respectively.

Analytical detection was performed using an Orbitrap Q Exactive Focus mass spectrometer (Thermo Fisher Scientific) with a heated electrospray ionization source. The mass spectrometer was operated in parallel reaction monitoring mode. Data were analyzed using the TraceFinder software (Thermo Fisher Scientific).

### 2.10 Statistical analysis

Statistical analyses were performed using JMP software version 13 (SAS Institute, Cary, NC, United States). Multiple comparisons were tested using the Tukey’s honestly significant difference test to compare the control, 2’-FL, and 2’-FL in combination with the M-63 group. Statistical significance was set at *p* < 0.05. Differences in the number of *B. infantis* before and after fermentation in the 2’-FL + M-63 group were analyzed using a student’s t-test. Statistical significance was set at *p* < 0.05.

## 3 Results

### 3.1 Prevalence of *Bifidobacterium* species in each participant

There was variation in the prevalence of *Bifidobacterium* species in the gut microbiota among the participants (Table 1). Among the infants, participants 1 and 3 had both *B. longum* and *B. breve*, whereas participant 2 had only *B. longum,* and participant 4 had both *B. breve* and *B. bifidum*. Among the young children, all four participants had *B. longum*, *B. breve* and *B. catenulatum* group. Only participant 5 had *B. bifidum,* and participant 7 had *B. adolescentis* group, respectively. None of the infants or young children had *B. infantis.* Cell number of each bifidobacterial species were shown as a heat map (Supplementary Figure S1). The gut microbiota of young children contained a higher number of bacterial genera compared to that of infants (Supplementary Figures S2A,B).

### 3.2 Effects of 2’-FL and M-63 on the microbiota composition in fecal fermentation

**Figure 1:**
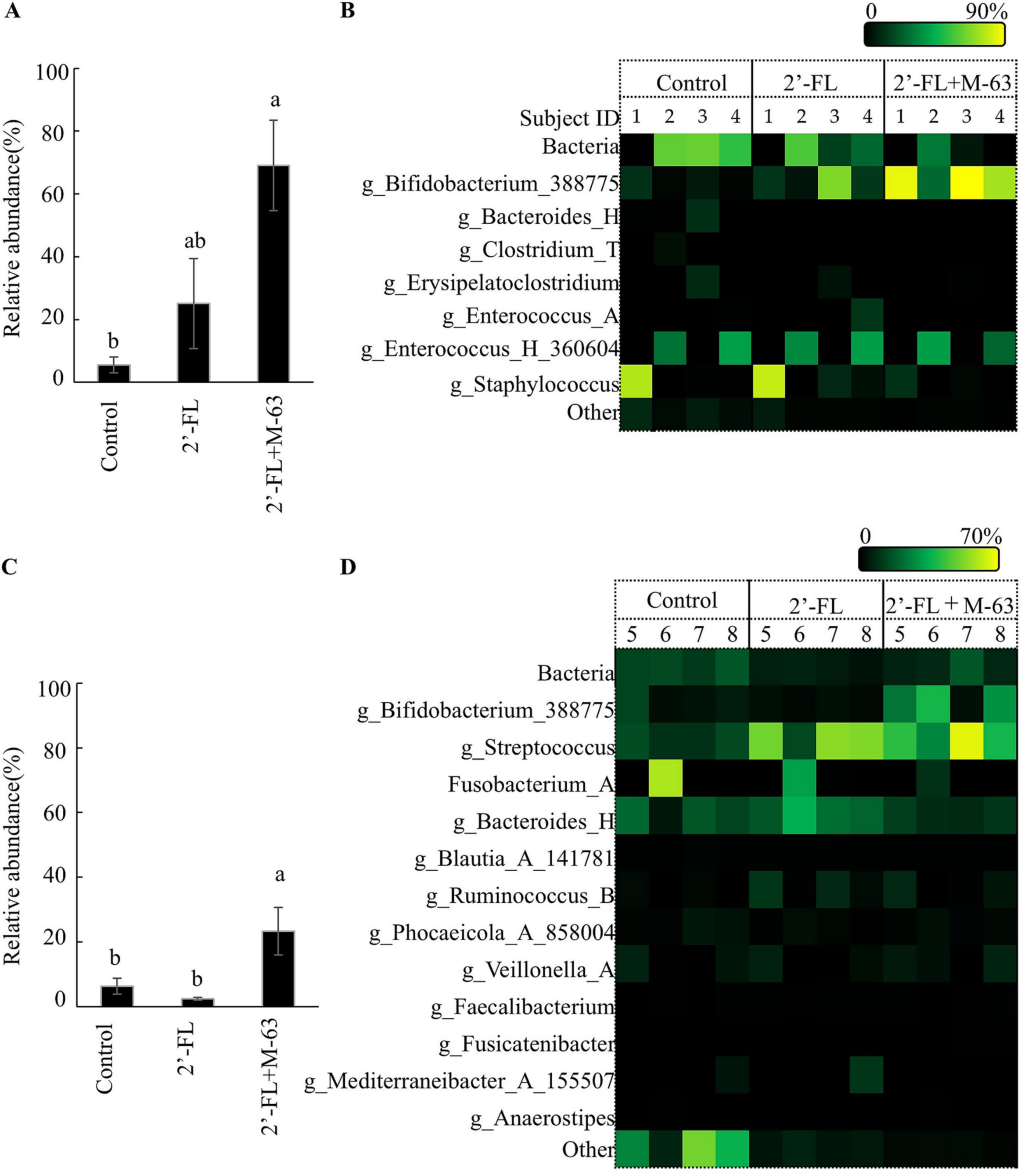
Effects of 2’-FL and M-63 on the composition (%) of infant and young children’s gut microbiota in pH-controlled single-batch fermentation. **(A)** Average relative abundance of *Bifidobacterium* in infants. **(B)** Composition of the fecal microbiota of each infant after fermentation. **(C)** Average abundance of *Bifidobacterium* in young children. **(D)** Composition of the fecal microbiota of each young child after fermentation. Data are expressed as means (*n* = 4) with SE. Different letters indicate significant differences (*p* < 0.05). 2’-FL, 2’-Fucosyllactose; M-63, *Bifidobacterium longum* subsp*. infantis* M-63.

In the fermentation with infant feces, supplementation with 2’-FL resulted in a trend toward increased relative abundance of *Bifidobacterium* compared to that in the control group (Figure 1A, *p* = 0.498), but without statistical significance. Heatmap analysis revealed significant inter-individual variability in the bifidogenic effects of 2’-FL; for instance, participant 3 responded well, whereas participant 2 exhibited no response (Figure 1B) regarding *Bifidobacterium*. Conversely, the 2’-FL + M-63 group showed a significant increase in the relative abundance of *Bifidobacterium* compared to the control group (*p* = 0.010) (Figure 1A). Heatmap analysis revealed that despite varying degrees, the relative abundance of *Bifidobacterium* increased in the fecal fermentation in all four infants (Figure 1B). No notable changes were observed in bacterial genera other than *Bifidobacterium* in either group (Figure 1B).

In the fermentation with the feces of young children, supplementation with 2’-FL resulted in no difference in the relative abundance of *Bifidobacterium* compared to that in the control group (Figure 1C); there was no change in the relative abundance of *Bifidobacterium* (Figure 1D). Conversely, 2’-FL + M-63 significantly increased the relative abundance of *Bifidobacterium* (Figure 1C; *p* < 0.05). Focusing on the inter-individual variability of the response, an increase in *Bifidobacterium* was observed in three out of four young children, but not in participant 7 (Figure 1D). Overall, the relative abundance of *Bifidobacterium* was lower in young children than in infants (Figures 1A–D).

Among genera other than *Bifidobacterium*, *Streptococcus* was significantly increased in both the 2’-FL and 2’-FL + M-63 groups, whereas *Bacteroides* was slightly increased in the 2’-FL group (Figure 1D; Supplementary Figure S2C). In the 2’-FL + M-63 group, increased M-63 after cultivation was confirmed in both the infants and young children through quantitative real-time PCR targeting *B. infantis* (Supplementary Figures S2D,E, *p* < 0.05).

### 3.3 Effects of 2’-FL and M-63 on SCFA and lactate production

**Figure 2:**
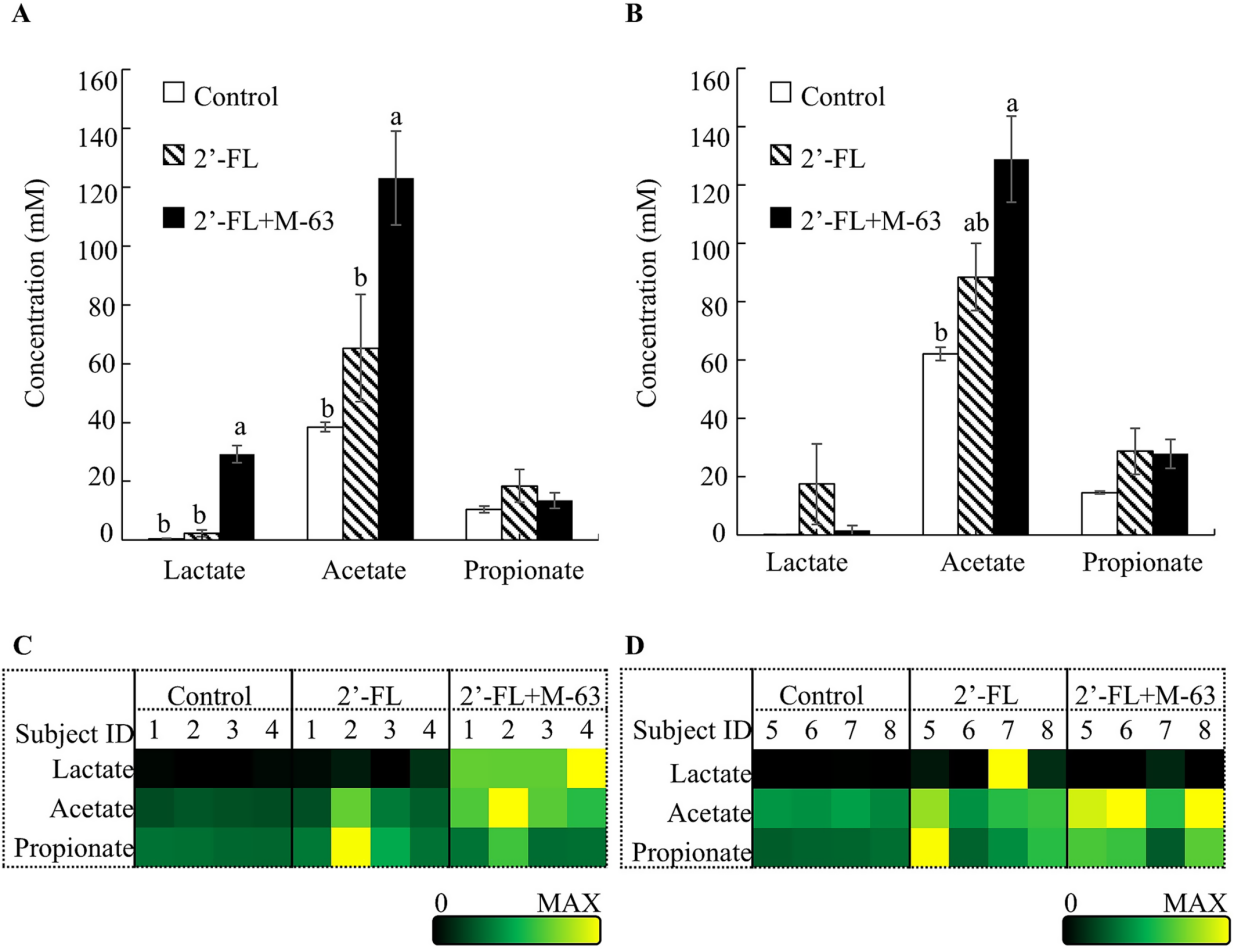
Effects of 2’-FL and M-63 on SCFAs and lactate production in fecal fermentation of infants and young children. **(A)** Concentrations of SCFAs and lactate in infant fecal fermentation. **(B)** Concentration of SCFAs and lactate in fecal fermentation of young children. **(C)** Heatmap showing SCFA and lactate concentrations for each participant in infant fecal fermentation **(D)** Heatmap showing SCFA and lactate concentrations for each participant in young children fecal fermentation. Data are expressed as mean ± SE (*n* = 4) **(A,B)**. Different letters indicate statistically significant differences (*p* < 0.05). 2’-FL, 2’-Fucosyllactose; M-63, *Bifidobacterium longum* subsp. *infantis* M-63.

In the fermentation with infant feces, the production of acetate and lactate was significantly increased in the 2’-FL + M-63 group compared to that in the control group, but not in the 2’-FL group (Figure 2A). Heatmap analysis showed inter-individual variability in acetate production in the 2’-FL group; a moderate increase in acetate was observed only in participant 2 among the 2’-FL group, whereas a moderate-to-high increase in acetate was observed in all four infants in the 2’-FL + M-63 group (Figure 2C). Consistent with this observation, 2’-FL in the culture media was completely consumed in the 2’-FL + M-63 group, whereas more than 80% of 2’-FL remained in the 2’-FL group after cultivation (Supplementary Figure S3; 80% in participants 1 and 4, 94% in participant 3, and 100% in participant 2).

In the fermentation with feces of young children, acetate production was significantly increased in the 2’-FL + M-63 group compared to that in the control group, but not in the 2’-FL group (Figure 2B). No significant differences were observed in lactate production among the three groups (Figure 2B). A slight-to-moderate increase in acetate was observed in the 2’-FL group, except for participant 6, whereas a moderate-to-high increase in acetate was observed in all four children in the 2’-FL + M-63 group (Figure 2D). Consistent with these results, 2’-FL in the culture media was well consumed in both the 2’-FL and 2’-FL + M-63 groups, except for participant 6 in the 2’-FL group (Supplementary Figure S3). There was no difference in propionate levels between the groups in the fermentations with either infants or young children (Figures 2A–D). Butyrate was not detected in the samples from either infants or young children.

As shown in Supplementary Figures S1, S3, the residual amount of 2’-FL in the medium of the 2’-FL group varied, even when the coexisting bifidobacterial species were the same or when the initial cell numbers of each bifidobacterial species were nearly equivalent (comparing participants 1and 3, and comparing participants 6 and 8, for example).

### 3.4 Effects of 2’-FL and M-63 on ALA production

**Figure 3:**
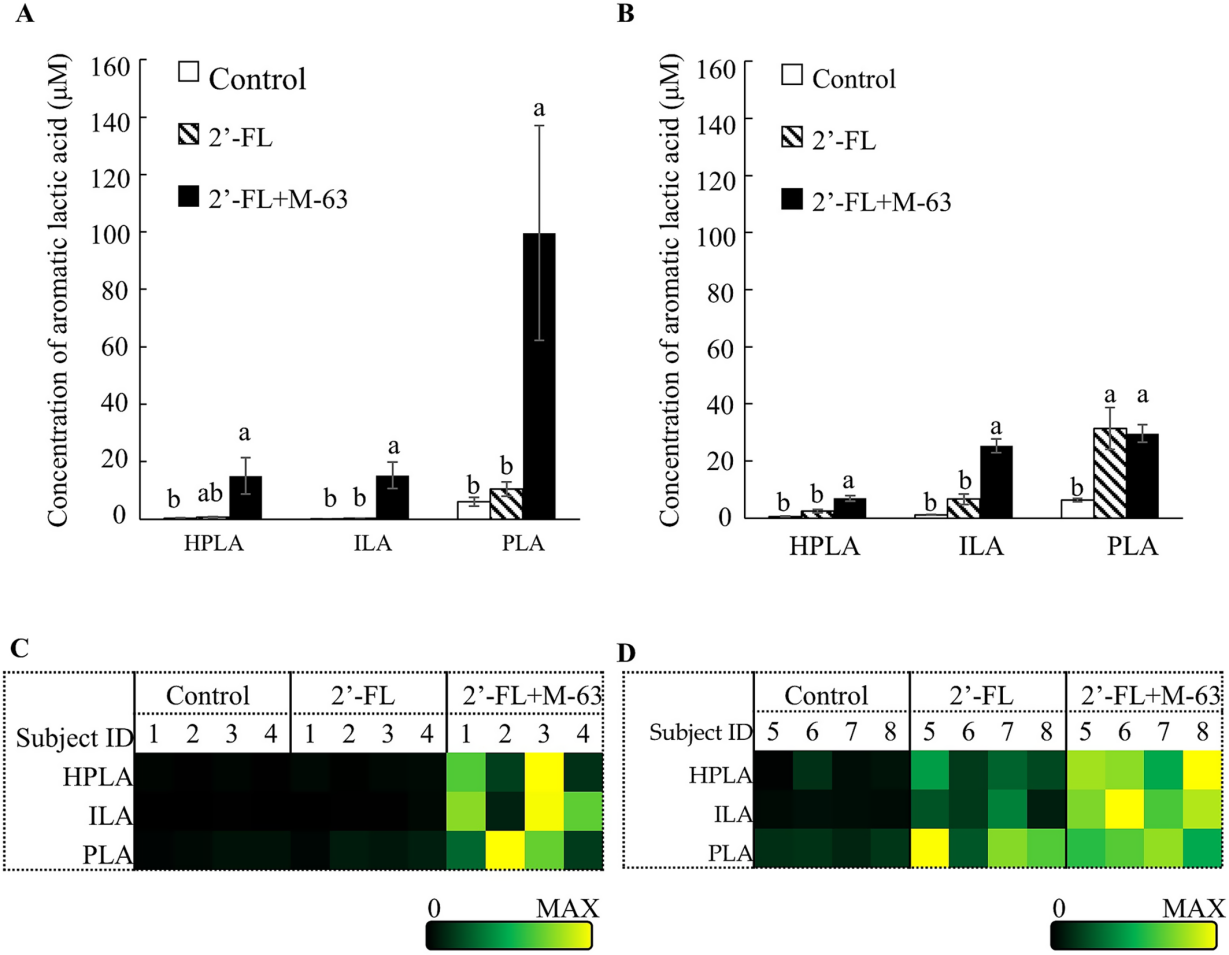
Effects of 2’-FL and M-63 on aromatic lactic acid production in infant fecal fermentation and young children. **(A)** Concentrations of aromatic lactic acids in infant fecal fermentation. **(B)** Concentrations of aromatic lactic acids in fecal fermentation of young children. **(C)** Heatmap showing aromatic lactic acids concentrations for each participant in infant fecal fermentation. **(D)** Heatmap showing aromatic lactic acids concentrations for each participant in young children fecal fermentation. Data are expressed as mean ± SE (*n* = 4) **(A,B)**. Different letters indicate statistically significant differences (*p* < 0.05). 2’-FL, 2’-Fucosyllactose; M-63, *Bifidobacterium longum* subsp. *infantis* M-63; HPLA, 4-hydroxyphenyllactic acid; ILA, indole-3-lactic acid; PLA, phenyllactic acid.

In the fermentation with infant feces, the production of ALAs was not affected by the supplementation of 2’-FL (Figure 3A). By contrast, the production of 4-hydroxyphenyllactic acid (HPLA), indole-3-lactic acid (ILA), and phenyllactic acid (PLA) was significantly enhanced in the 2’-FL + M-63 group compared to that in the control group (Figure 3A). Nevertheless, a large degree of individual variability was observed in the production of these ALAs in the 2’-FL + M-63 group (Figure 3C).

In the fermentation with feces of young children, the production of PLA was increased in the 2’-FL group. However, the production of all three ALAs (HPLA, ILA, and PLA) were increased in the 2’-FL + M-63 group compared to that in the control group (Figure 3B). Inter-individual variability was greater in the 2’-FL group than in the 2’-FL + M-63 group for all three ALAs (Figure 3D). Focusing on the 2’-FL + M-63 group, inter-individual variability was smaller in the fermentation of young children than in that of infants (Figures 3C,D).

## 4 Discussion

The physiological benefits of HMOs are, at least partly, mediated by metabolites such as acetate and ALAs, produced during the utilization of HMOs by infant-type HRB. Nevertheless, the effects of HMOs on the gut microbiota of infants who lack HRB capable of utilizing HMOs, or on children over 3 years old after the decrease of infant-type HRB, have not been sufficiently investigated. This study is the first to address this issue.

In this study, the detected *Bifidobacterium* species were primarily *B. longum* and *B. breve* in infants and *B. longum*, *B. breve* and *B. catenulatum* group in young children. Nevertheless, there was variation in the prevalence of *Bifidobacterium* species in the gut microbiota among participants, as observed in previous reports (21, 25). Specifically, all infants had either *B. longum* or *B. breve,* alone or both, and one infant had *B. bifidum*. All young children had *B. longum, B. breve*, and *B. catenulatum* group, with one individual additionally carrying *B. bifidum* and another having *B. adolescentis* group. Given that HMO-utilization pathways in bifidobacteria vary at the species or even strain level (14), such individual variations in the prevalence of *Bifidobacterium* species are thought to be related to differences in the response to HMO among individuals. In our experiment, the individual differences in responsiveness to 2’-FL among the 2’-FL only group are most likely due to strain-level differences rather than differences in species or initial cell numbers, as indicated by the data of species composition, initial cell numbers and residual amount of 2’-FL.

In this study, fermentation with the feces of these infants supplemented with 2’-FL led to a significant difference in response. One infant showed a significant increase in bifidobacteria, two infants showed a slight increase, and one infant showed almost no response. Reflecting this, approximately 80% of 2’-FL in the culture media remained unconsumed. Additionally, there was considerable individual variation in acetate production, and ALA production was not observed in any of the four infants. This indicates a notable individual variability in the response to 2’-FL. In contrast, the combination of 2’-FL and M-63 resulted in the proliferation of bifidobacteria and promoted the production of acetate and ALAs in all four infants. Correspondingly, 2’-FL in the fermentation media was completely consumed. Notably, all the infants in this study were breastfed. This indicates that, even among breastfed infants, not all have *Bifidobacterium* capable of utilizing 2’-FL, and suggesting that these infants do not fully benefit from 2’-FL. Furthermore, the co-administration of infant-type HRB with a high HMO-utilizing capacity, such as *B. infantis* M-63 (13), could be an effective solution to this issue.

In the fermentation with the feces of young children, an increase in bifidobacteria was not observed with supplementation of 2’-FL. Despite the presence of bifidobacterial species, such as *B. longum*, *B. breve*, and *B. bifidum* in the feces from these young children, it is intriguing that 2’-FL did not exhibit any bifidogenic effect. This finding reaffirms the interspecies and inter-strain variations in the HMO-utilizing capacity of bifidobacteria within the same species. Furthermore, it also suggests that a shift from strains with high HMO-utilizing capacity to those with lower capacity within the same species occurs during the transition from infants to young children, marked by the cessation of breastfeeding. This hypothesis could be confirmed by a longitudinal analysis of bifidobacterial strains from the gut of individuals from birth to beyond 3 years of age, focusing on their HMO-utilizing capacity.

HMOs, including 2’-FL, can be utilized by certain bacteria other than bifidobacteria, such as *Bacteroides* and *Streptococcus* species (33–38). In the 2’-FL-only group of the young children, an increase in *Streptococcus* and *Bacteroides* was observed accompanied with consumption of the added 2’-FL. This suggests that 2’-FL can be utilized by these bacteria (33–38). Conversely, in the 2’-FL + M-63 group, a bifidogenic effect and the promotion of acetate and ALA production were observed in young children. Correspondingly, the added 2’-FL was completely consumed. This suggests that, as with infants, the co-administration of infant-type HRB with a high HMO-utilizing capacity alongside 2’-FL is effective in providing the benefits of 2’-FL to young children beyond the age of 3. However, the impact for young children was slightly weaker compared to that for infants. In the 2’-FL + M-63 group, although the occupancy rate of bifidobacteria reached nearly 70% in infants, it was only about 20% in young children. This may be due to potential competition between M-63 and other bacteria capable of utilizing 2’-FL in the gut of young children, such as *Bacteroides* or *Streptococcus*, or because the gut microbial environment of young children may not be suitable for bifidobacteria to utilizing 2’-FL. Clarifying this point in future studies is expected to provide a comprehensive view of HMO metabolism in the guts of infants and young children and insights into the adaptation of the gut microbiota to dietary prebiotics.

Butyrate was not detected in the present experiment. Although it is known that butyrate concentrations are low in the gut of infants (39), the absence of detectable butyrate even in young children may be partly due to the limited proliferation of butyrate-producing bacterial genera such as *Blautia* and *Faecalibacterium,* in the experimental conditions of this study.

Notably, *B. infantis* was absent in all participants, regardless of age. This aligns with a recent report indicating a lower detection frequency of *B. infantis* in infants from industrialized countries than in those from developing countries (20, 40–42). The prevalence of *B. infantis* in infants was 7.4% in a U. S. cohort under 2 years of age (43), 0–14.3% in an Australian cohort of eight-week-old infants (40), and similar results have been reported in pre-weaning infants in a Japanese study (24). Considering the positive impact of *B. infantis* on immune development of infants (44), its absence raises concerns about its potential negative effects on infant health. The positive effects of *B. infantis* on immune development are partly mediated by the production of ALAs, including ILA (23, 44). Among ALAs, ILA, an indole derivative, is particularly notable for its ability to suppress inflammation via AhR (45, 46), and suppress Th2 and Th17 cells via galectin-1 (44). Furthermore, recent reports indicate that the prevalence of *Bifidobacterium* producing ALAs during the neonatal period, particularly HPLA, was inversely associated with the development of food allergen-specific IgE (47). Based on the results of this study and previous reports, co-administering *B. infantis* with HMOs could be a useful strategy for restoring a *B. infantis*-enriched gut bifidobacterial community (42) and increasing the health benefits of both HMOs and bifidobacteria. Future clinical studies are needed to confirm the efficacy of this approach in enhancing ILA production and supporting immune development in infants and young children.

The increase in *Streptococcus* and *Bacteroides* observed in young children due to 2’-FL was an unintended increase, although these bacteria are not necessarily harmful. In other words, it was reconfirmed that when there are few Bifidobacteria capable of utilizing 2’-FL, unintended bacterial populations may proliferate, and resulting dysbiosis could occur. In this context, *Clostridium perfringens*, considered a pathobiont species in preterm infants, can also utilize 2’-FL to proliferate; however, its proliferation and alpha-toxin production are suppressed in the presence of infant-type HRB (48). Based on these findings, when formulating 2’-FL into infant and young child formula, it is considered desirable to include *Bifidobacterium* strains capable of utilizing 2’-FL, not only for functional benefits but also for safety reasons.

In summary, the results of this study indicate the presence of “non-responders” to 2’-FL, in whom the gut *Bifidobacterium* population is not increased by 2’-FL, in both infants and young children aged 3–5 years. The study suggests that: (i) this tendency is particularly pronounced in young children over the age of 3, especially after the cessation of breastfeeding, and (ii) co-administration of *B. infantis* M-63 with 2’-FL can convert these “non-responders” into “responders.” These observations regarding 2’-FL and M-63 are consistent with a previous clinical trial which found that the number of *Bifidobacterium* in the gut of infants administered M-63 correlated with the frequency of breastfeeding, suggesting that at least some of the effects of M-63 are related to the utilization of HMOs from human milk (24). Taken together, these results suggest that to ensure that infants and young children receive the full health benefits of HMOs, it is crucial to have *Bifidobacterium* with high HMO-utilization capacity, such as *B. infantis*, in the gut. In the absence of these bacteria, oral supplementation with probiotic strains may be an effective strategy.

This study has some limitations. Firstly, due to the hardware limitations of our instrument and the experimental challenges associated with handling a large number of samples, the sample size was small, consisting of only four infants and four young children. Secondly, as the culture model used in this study did not include host cells, it was unable to replicate the complex interactions between the host and gut microbiota, such as modulation by the host immune system or antimicrobial peptides. Thirdly, the study only used 2’-FL while HMOs are highly diverse in structure and did not consider the potential synergistic effects between different HMOs. Therefore, this model cannot fully replicate the *in vivo* responses. Based on these limitations, this is a “proof-of-concept” study and further clinical trials are needed to examine the efficacy of 2’-FL or other HMOs, both alone and in combination with M-63.

In conclusion, this study is the first to demonstrate that the health benefits from 2’-FL may be restricted by inter-individual and age-dependent differences of the response of gut microbiota, and supplementation of HMO-high-utilizable probiotic *B. infantis* could overcome this restriction. These findings may provide new insights for the improvement of infant and young child formulae.

## Data Availability

The raw data supporting the conclusions of this article are available from the corresponding author upon reasonable request.

## References

[1] Soyyılmaz B Mikš MH Röhrig CH Matwiejuk M Meszaros-Matwiejuk A Vigsnæs LK. The mean of milk: a review of human milk oligosaccharide concentrations throughout lactation. Nutrients. (2021) 13:2737. doi: 10.3390/nu13082737, 34444897 PMC8398195

[2] Urashima T Asakuma S Leo F Fukuda K Messer M Oftedal OT. The predominance of type I oligosaccharides is a feature specific to human breast milk. Adv Nutr. (2012) 3:473S–82S. doi: 10.3945/an.111.001412, 22585927 PMC3649485

[3] Musilova S Rada V Vlkova E Bunesova V. Beneficial effects of human milk oligosaccharides on gut microbiota. Benef Microbes. (2014) 5:273–83. doi: 10.3920/BM2013.0080 24913838

[4] Jeong K Nguyen V Kim J. Human milk oligosaccharides: the novel modulator of intestinal microbiota. BMB Rep. (2012) 45:433–41. doi: 10.5483/BMBRep.2012.45.8.168 22917027

[5] Yu ZT Chen C Kling DE Liu B McCoy JM Merighi M . The principal fucosylated oligosaccharides of human milk exhibit prebiotic properties on cultured infant microbiota. Glycobiology. (2013) 23:169–77. doi: 10.1093/glycob/cws138, 23028202 PMC3531294

[6] Jacobs JP Lee ML Rechtman DJ Sun AK Autran C Niklas V. Human milk oligosaccharides modulate the intestinal microbiome of healthy adults. Sci Rep. (2023) 13:14308. doi: 10.1038/s41598-023-41040-5, 37652940 PMC10471580

[7] Chen Y Chen Z Zhu Y Wen Y Zhao C Mu W. Recent progress in human milk oligosaccharides and its antiviral efficacy. J Agric Food Chem. (2024) 72:7607–17. doi: 10.1021/acs.jafc.3c09460, 38563422

[8] Kim JY Lee S Kim G Shin HJ Lee EJ Lee CS . Ameliorating effect of 2′-fucosyllactose and 6′-sialyllactose on lipopolysaccharide-induced intestinal inflammation. J Dairy Sci. (2024) 107:4147–60. doi: 10.3168/jds.2024-24325, 38490539

[9] Parschat K Melsaether C Jäpelt KR Jennewein S. Clinical evaluation of 16-week supplementation with 5HMO-mix in healthy-term human infants to determine tolerability, safety, and effect on growth. Nutrients. (2021) 13:2871. doi: 10.3390/nu13082871, 34445031 PMC8401119

[10] Bosheva M Tokodi I Krasnow A Pedersen HK Lukjancenko O Eklund AC . Infant formula with a specific blend of five human milk oligosaccharides drives the gut microbiota development and improves gut maturation markers: a randomized controlled trial. Front Nutr. (2022) 9:920362. doi: 10.3389/fnut.2022.920362, 35873420 PMC9298649

[11] Falsaperla R Sortino V Gambilonghi F Vitaliti G Striano P. Human milk oligosaccharides and their pivotal role in gut-brain axis modulation and neurologic development: a narrative review to decipher the multifaceted interplay. Nutrients. (2024) 16:3009. doi: 10.3390/nu16173009 39275324 PMC11397282

[12] Walsh C Lane JA van Sinderen D Hickey RM. Human milk oligosaccharides: shaping the infant gut microbiota and supporting health. J Funct Foods. (2020) 72:104074. doi: 10.1016/j.jff.2020.104074, 32834834 PMC7332462

[13] Thongaram T Hoeflinger JL Chow JM Miller MJ. Human milk oligosaccharide consumption by probiotic and human-associated bifidobacteria and lactobacilli. J Dairy Sci. (2017) 100:7825–33. doi: 10.3168/jds.2017-12753, 28780103

[14] Katayama T. Host-derived glycans serve as selected nutrients for the gut microbe: human milk oligosaccharides and bifidobacteria. Biosci Biotechnol Biochem. (2016) 80:621–32. doi: 10.1080/09168451.2015.1132153, 26838671

[15] Sakurai T Odamaki T Xiao JZ. Production of indole-3-lactic acid by *Bifidobacterium* strains isolated from human infants. Microorganisms. (2019) 7:340. doi: 10.3390/microorganisms7090340, 31514325 PMC6780619

[16] Laursen MF Sakanaka M von Burg N Mörbe U Andersen D Moll JM . *Bifidobacterium* species associated with breastfeeding produce aromatic lactic acids in the infant gut. Nat Microbiol. (2021) 6:1367–82. doi: 10.1038/s41564-021-00970-4, 34675385 PMC8556157

[17] Huda MN Ahmad SM Alam MJ Khanam A Kalanetra KM Taft DH . *Bifidobacterium* abundance in early infancy and vaccine response at 2 years of age. Pediatrics. (2019) 143:e20181489. doi: 10.1542/peds.2018-1489 30674610 PMC6361348

[18] Barratt MJ Nuzhat S Ahsan K Frese SA Arzamasov AA Sarker SA . *Bifidobacterium infantis* treatment promotes weight gain in Bangladeshi infants with severe acute malnutrition. Sci Transl Med. (2022) 14:eabk1107. doi: 10.1126/scitranslmed.abk1107 35417188 PMC9516695

[19] Batta VK Rao SC Patole SK. *Bifidobacterium infantis* as a probiotic in preterm infants: a systematic review and meta-analysis. Pediatr Res. (2023) 94:1887–905. doi: 10.1038/s41390-023-02716-w, 37460707 PMC10665187

[20] Xu J Duar RM Quah B Gong M Tin F Chan P . Delayed colonization of *Bifidobacterium* spp. and low prevalence of *B. infantis* among infants of Asian ancestry born in Singapore: insights from the GUSTO cohort study. Front Pediatr. (2024) 12:1421051. doi: 10.3389/fped.2024.1421051, 38915873 PMC11194334

[21] Kato K Odamaki T Mitsuyama E Sugahara H Xiao JZ Osawa R. Age-related changes in the composition of gut *Bifidobacterium* species. Curr Microbiol. (2017) 74:987–95. doi: 10.1007/s00284-017-1272-4, 28593350 PMC5486783

[22] Odamaki T Kato K Sugahara H Hashikura N Takahashi S Xiao JZ . Age-related changes in gut microbiota composition from newborn to centenarian: a cross-sectional study. BMC Microbiol. (2016) 16:90. doi: 10.1186/s12866-016-0708-5, 27220822 PMC4879732

[23] Wong CB Huang H Ning Y Xiao J. Probiotics in the new era of human milk oligosaccharides (HMOs): HMO utilization and beneficial effects of *Bifidobacterium longum* subsp. *infantis* M-63 on infant health. Microorganisms. (2024) 12:1014. doi: 10.3390/microorganisms12051014, 38792843 PMC11124435

[24] Hiraku A Nakata S Murata M Xu C Mutoh N Arai S . Early probiotic supplementation of healthy term infants with *Bifidobacterium longum* subsp. *infantis* M-63 is safe and leads to the development of *Bifidobacterium*-predominant gut microbiota: a double-blind, placebo-controlled trial. Nutrients. (2023) 15:1402. doi: 10.3390/nu15061402, 36986131 PMC10055625

[25] Mingat SX Ehara T Nakamura H Miyaji K. Comparative study of prebiotics for infants using a fecal culture system: insights into responders and non-responders. Nutrients. (2024) 16:3347. doi: 10.3390/nu16193347, 39408314 PMC11478422

[26] Matsuki T Watanabe K Fujimoto J Kado Y Takada T Matsumoto K . Quantitative PCR with 16S rRNA-gene-targeted species-specific primers for analysis of human intestinal bifidobacteria. Appl Environ Microbiol. (2004) 70:167–73. doi: 10.1128/AEM.70.1.167-173.2004, 14711639 PMC321263

[27] Matsuki T Watanabe K Tanaka R Oyaizu H. Rapid identification of human intestinal bifidobacteria by 16S rRNA-targeted species- and group-specific primers. FEMS Microbiol Lett. (1998) 167:113–21. doi: 10.1111/j.1574-6968.1998.tb13216.x, 9809413

[28] Toda K Hisata K Satoh T Katsumata N Odamaki T Mitsuyama E . Neonatal oral fluid as a transmission route for bifidobacteria to the infant gut immediately after birth. Sci Rep. (2019) 9:8692. doi: 10.1038/s41598-019-45198-9, 31213639 PMC6582144

[29] Ejima R Mishima R Sen A Yamaguchi K Mitsuyama E Kaneko H . The impact of fermented milk products containing *Bifidobacterium longum* BB536 on the gut environment: a randomized double-blind placebo-controlled trial. Nutrients. (2024) 16:3580. doi: 10.3390/nu16213580, 39519413 PMC11547261

[30] Bolyen E Rideout JR Dillon MR Bokulich NA Abnet CC Al-Ghalith GA . Reproducible, interactive, scalable and extensible microbiome data science using QIIME 2. Nat Biotechnol. (2019) 37:852–7. doi: 10.1038/s41587-019-0209-9, 31341288 PMC7015180

[31] Callahan BJ McMurdie PJ Rosen MJ Han AW Johnson AJ Holmes SP. DADA2: high-resolution sample inference from Illumina amplicon data. Nat Methods. (2016) 13:581–3. doi: 10.1038/nmeth.3869, 27214047 PMC4927377

[32] Morozumi M Izumi H Tsuda M Tabata F Nakamura H Miyaji K . Changes in and relationships between human milk oligosaccharides and microRNAs in milk-derived extracellular vesicles during the first 4 months of lactation. Front Nutr. (2025) 12:1694093. doi: 10.3389/fnut.2025.1694093, 41496911 PMC12766977

[33] Marcobal A Barboza M Sonnenburg ED Pudlo N Martens EC Desai P . *Bacteroides* in the infant gut consume milk oligosaccharides via mucus-utilization pathways. Cell Host Microbe. (2011) 10:507–14. doi: 10.1016/j.chom.2011.10.007, 22036470 PMC3227561

[34] Kijner S Cher A Yassour M. The infant gut commensal *Bacteroides dorei* presents a generalized transcriptional response to various human milk oligosaccharides. Front Cell Infect Microbiol. (2022) 12:854122. doi: 10.3389/fcimb.2022.854122, 35372092 PMC8971754

[35] Zhou Y Liu X Chen H Zhao J Zhang H Chen W . Isolation and characterisation of *Streptococcus* spp. with human milk oligosaccharides utilization capacity from human milk. Foods. (2024) 13:1291. doi: 10.3390/foods13091291, 38731662 PMC11083076

[36] Yu ZT Chen C Newburg DS. Utilization of major fucosylated and sialylated human milk oligosaccharides by isolated human gut microbes. Glycobiology. (2013) 23:1281–92. doi: 10.1093/glycob/cwt065, 24013960 PMC3796377

[37] Marcobal A Sonnenburg JL. Human milk oligosaccharide consumption by intestinal microbiota. Clin Microbiol Infect. (2012) 18:12–5. doi: 10.1111/j.1469-0691.2012.03863.x, 22647041 PMC3671919

[38] Cho S Samuel TM Li T Howell BR Baluyot K Hazlett HC . Interactions between *Bifidobacterium* and *Bacteroides* and human milk oligosaccharides and their associations with infant cognition. Front Nutr. (2023) 10:1216327. doi: 10.3389/fnut.2023.1216327, 37457984 PMC10345227

[39] Tsukuda N Yahagi K Hara T Watanabe Y Matsumoto H Mori H . Key bacterial taxa and metabolic pathways affecting gut short-chain fatty acid profiles in early life. ISME J. (2021) 15:2574–90. doi: 10.1038/s41396-021-00937, 33723382 PMC8397723

[40] Lawley B Munro K Hughes A Hodgkinson AJ Prosser CG Lowry D . Differentiation of *Bifidobacterium longum* subspecies *longum* and *infantis* by quantitative PCR using functional gene targets. PeerJ. (2017) 5:e3375. doi: 10.7717/peerj.3375, 28560114 PMC5446769

[41] Olm MR Dahan D Carter MM Merrill BD Yu FB Jain S . Robust variation in infant gut microbiome assembly across a spectrum of lifestyles. Science. (2022) 376:1220–3. doi: 10.1126/science.abj2972, 35679413 PMC9894631

[42] Insel RA Jarman JB Torres PJ Van Dien S Culler SJ de Vos WM. Restoring a gut *Bifidobacterium* community in early infancy. Cell Host Microbe. (2025) 33:2012–6. doi: 10.1016/j.chom.2025.10.017, 41380667

[43] Tso L Bonham KS Fishbein A Rowland S Klepac-Ceraj V. Targeted high-resolution taxonomic identification of *Bifidobacterium longum* subsp. *infantis* using human milk oligosaccharide metabolizing genes. Nutrients. (2021) 13:2833. doi: 10.3390/nu13082833, 34444993 PMC8401031

[44] Henrick BM Rodriguez L Lakshmikanth T Pou C Henckel E Arzoomand A . Bifidobacteria-mediated immune system imprinting early in life. Cell. (2021) 184:3884–3898.e11. doi: 10.1016/j.cell.2021.05.030, 34143954

[45] Krishnan S Ding Y Saeidi N Choi M Sridharan GV Sherr DH . Gut microbiota-derived tryptophan metabolites modulate inflammatory response in hepatocytes and macrophages. Cell Rep. (2018) 23:1099–111. doi: 10.1016/j.celrep.2018.03.109, 29694888 PMC6392449

[46] Qian X. Li Q. Zhu H. Chen Y. Lin G. Zhang H. . Bifidobacteria with indole-3-lactic acid-producing capacity exhibit psychobiotic potential via reducing neuroinflammation. Cell Rep Med. (2024) 5:101798. doi:10.1016/j.xcrm.2024.101798, . 39471819 PMC11604549

[47] Myers PN Dehli RK Mie A Moll JM Roager HM Eriksen C . Early-life colonization by aromatic-lactate-producing bifidobacteria lowers the risk of allergic sensitization. Nat Microbiol. (2026) 11:429–41. doi: 10.1038/s41564-025-02244-9, 41526643

[48] Nakajima A Arzamasov AA Sakanaka M Murakami R Kozakai T Yoshida K . *In vitro* competition with *Bifidobacterium* strains impairs potentially pathogenic growth of *Clostridium perfringens* on 2′-fucosyllactose. Gut Microbes. (2025) 17:2478306. doi: 10.1080/19490976.2025.2478306, 40102238 PMC11956901

