# Supplementary material for: Synbiotic effects of 2’-fucosyllactose and Bifidobacterium longum subsp. infantis M-63 in fermented human fecal communities
Source: Front Nutr. 2026 May 14;13:1744839. doi: 10.3389/fnut.2026.1744839 (PMC13218082; doi:10.3389/fnut.2026.1744839)
Supplement: Supplementary file 1 [file Data_Sheet_1.pdf]

Supplementary Material

1 Supplementary Table 1

PCR primers and Annealing temperatures for *Bifidobacterium* species detection

Target	Primer	Sequences (5'-3')	Annealing temperature	References
Bifidobacterium longum subsp. *longum*	BiLON-1	TTCCAGTTGATCGCATGGTC	55 °C	(26)
	BiLON-2	GGGAAGCCGTATCTCTACGA		
Bifidobacterium breve	BiBRE-1	CCGGATGCTCCATCACAC	55 °C	(26)
	BiBRE-2	ACAAAGTGCCTTGCTCCCT		
Bifidobacterium bifidum	BiBIF-1	CCACATGATCGCATGTGATTG	55 °C	(27)
	BiBIF-2	CCGAAGGCTTGCTCCCAAA		
Bifidobacterium longum subsp. *infantis*	INF clpC-F	ACATCCAGGACCGTAACCTG	60 °C	(28)
	INF clpC-R	GCTTGTGCAGCTCCGTCT		
Bifidobacterium adolescentis group	BiADOg-1a	CTCCAGTTGGATGCATGTC	55 °C	(26)
	BiADOg-1b	TCCAGTTGACCGCATGGT		
	BiADOg-2	CGAAGGCTTGCTCCCAGT		
Bifidobacterium catenulatum group	BiCATg-1	CGGATGCTCCGACTCCT	55 °C	(26)
	BiCATg-2	CGAAGGCTTGCTCCCGAT		

2 Supplementary Figure 1

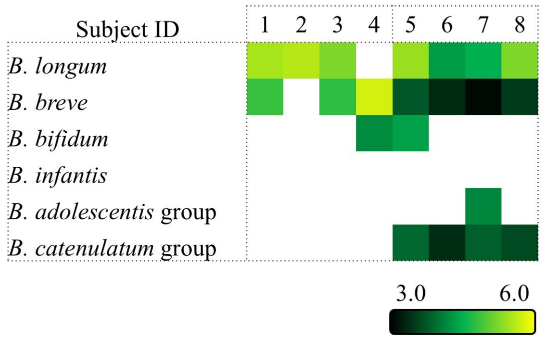

Supplementary Figure 1. Heat map of initial cell number of each bifidobacterial species. Bacterial counts in 1ml medium before cultivation were quantified by real-time PCR and log_{10}-transformed prior to heat map visualization.

3 Supplementary Figure 2

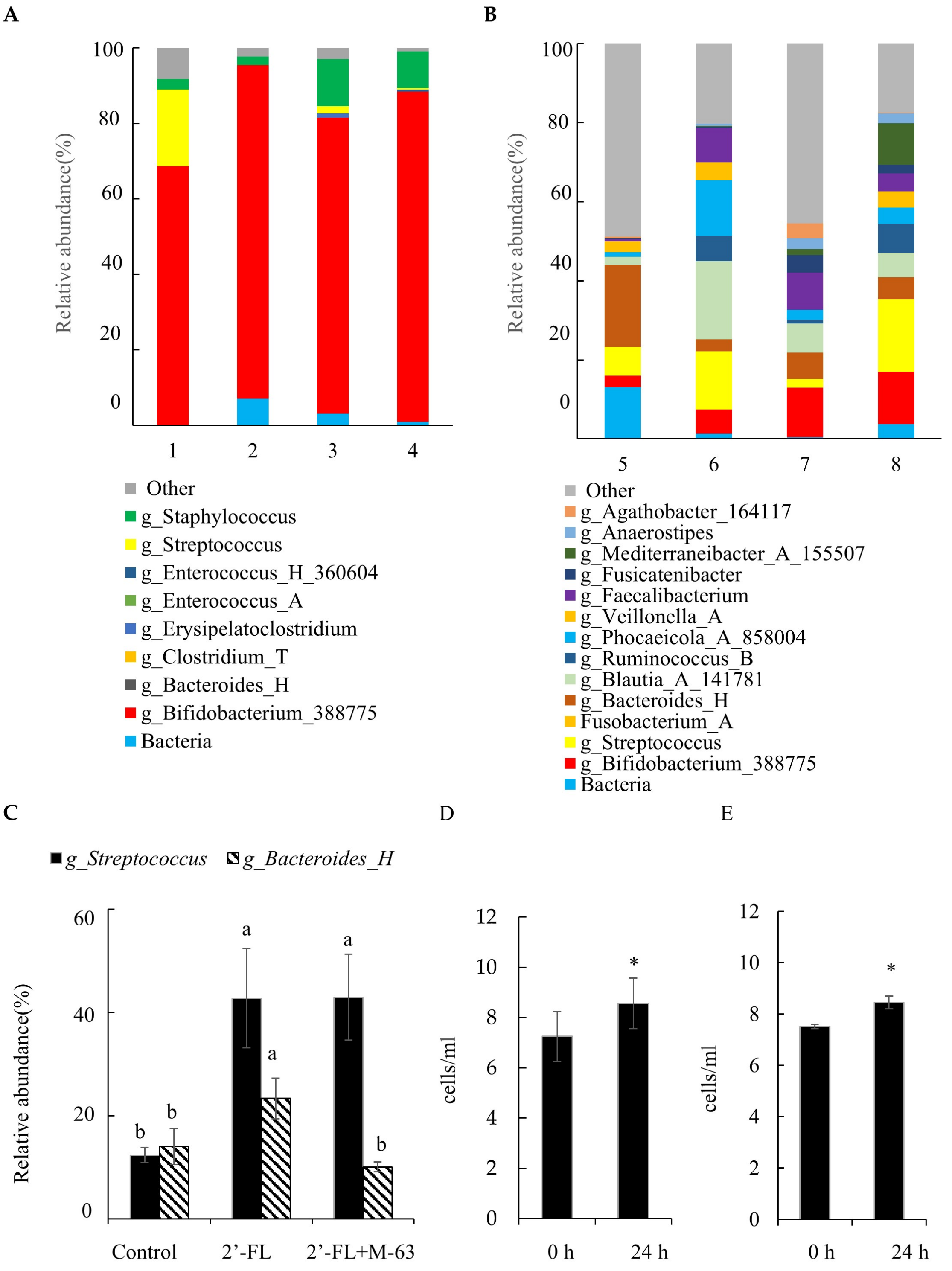

Supplementary Figure 2. Microbial profiles and *B. infantis* levels in infant and young child fecal samples pre- and post-fermentation. (A) Relative abundance of fecal microbiota in each infant sample before fermentation. (B) Relative abundance of fecal microbiota in samples from each young child before fermentation. (C) Relative abundance of *Streptococcus* and *Bacteroides* spp. in the fecal fermentation of young children. (D) Number of *B. infantis* in the fecal fermentation of infants before and after fermentation in 2’-FL+M-63 group. (E) Number of *B. infantis* in the fecal fermentation of young children before and after fermentation in 2’-FL+M-63 group. Data are expressed as the mean ($n = 4$) with SE (C, D, E). Different letters indicate significance ($p < 0.05$) in panel C. * indicates a significant difference ($p < 0.05$) at 24 h compared with 0 h in panels D and E.

4 Supplementary Figure 3

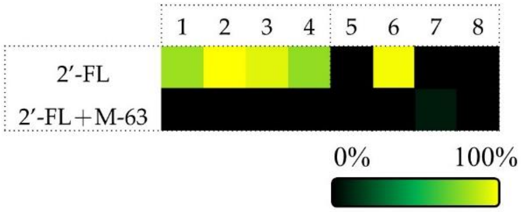

Supplementary Figure 3. Residual amount of 2’-FL after fermentation. Heatmap of residual 2’-FL after fermentation in fecal fermentation of infants (1-4) and young children (5–8).
